# Maternal-fetal cytokine profiles in acute SARS-CoV-2 “breakthrough” infection after COVID-19 vaccination

**DOI:** 10.3389/fimmu.2024.1506203

**Published:** 2025-01-08

**Authors:** Claire H. Packer, Olyvia Jasset, Nikolina Hanniford, Sara Brigida, Stepan Demidkin, Roy H. Perlis, Andrea G. Edlow, Lydia L. Shook

**Affiliations:** ^1^ Department of Obstetrics and Gynecology, Massachusetts General Hospital, Harvard Medical School, Boston, MA, United States; ^2^ Department of Psychiatry, Massachusetts General Hospital, Harvard Medical School, Boston, MA, United States; ^3^ Center for Genomic Medicine, Massachusetts General Hospital, Boston, MA, United States; ^4^ Vincent Center for Reproductive Biology, Massachusetts General Hospital, Boston, MA, United States

**Keywords:** COVID-19, pregnancy, cytokines, COVID-19 vaccines, SARS-CoV-2, breakthrough infection

## Abstract

**Objective:**

Vaccination is protective against severe COVID-19 disease, yet whether vaccination reduces COVID-19-associated inflammation in pregnancy has not been established. The objective of this study is to characterize maternal and cord cytokine profiles of acute SARS-CoV-2 “breakthrough” infection (BTI) after vaccination, compared with unvaccinated infection and uninfected controls.

**Study design:**

66 pregnant individuals enrolled in the MGH COVID-19 biorepository (March 2020-April 2022) were included. Maternal sera were collected from 26 unvaccinated and 21 vaccinated individuals with acute SARS-CoV-2 infection. Cord sera were collected at delivery. Maternal and cord sera from 19 term dyads without current or prior SARS-CoV-2 infection were analyzed as controls. Cytokines were quantified using the Human Inflammation 20-Plex ProcartaPlex assay.

**Results:**

There was a significantly higher incidence of severe/critical maternal illness in unvaccinated pregnant individuals with SARS-CoV-2 compared to vaccinated (10/26 (38%) *vs*. 0/21 (0%), p<0.01). Significantly higher maternal levels of TNFα and CD62P were observed in vaccinated individuals with SARS-CoV-2 BTI compared with unvaccinated individuals with infection (p<0.05). Network correlation analyses revealed a distinct maternal cytokine response to SARS-CoV-2 in vaccinated *vs* unvaccinated individuals. Neither unvaccinated nor vaccinated SARS-CoV-2 infection resulted in elevated cord cytokines compared to controls. Multivariate analyses demonstrate distinct maternal and cord cytokine profiles in the setting of maternal SARS-CoV-2 at delivery.

**Conclusion:**

Vaccination was associated with higher maternal cytokine levels during acute SARS-CoV-2 infection compared to unvaccinated infection, which may reflect vaccine-mediated priming of the immune system. A fetal inflammatory response specific to maternal SARS-CoV-2 infection was not observed.

## Introduction

Multiple maternal exposures, ranging from viral and bacterial infection, to obesity, substance use, and stress, can activate the maternal immune system, which in turn can be associated with fetoplacental immune activation and downstream consequences for fetal developmental programming (1–6). The *in utero* mechanisms that differentially impact developmental trajectories in offspring exposed to maternal immune activation remain an important knowledge gap (7–9) However, multiple lines of evidence support the association between exposure and long-term neurodevelopmental and metabolic disease in offspring (10–14)

COVID-19 infection during pregnancy drives proinflammatory signatures in the maternal proteome and alters immune cell subsets and functions (15, 16). Innate antiviral immune responses to maternal SARS-CoV-2 infection have also been observed in the placenta, including upregulated interferon-stimulated genes and pro-inflammatory cytokines, even in the absence of direct placental infection (17–20). Limited evidence suggests the potential for maternal COVID-19 infection to stimulate an immune response in the fetus, with observations including upregulated classic proinflammatory cytokines IL-6, IL-8, and IP-10 (CXCL10) in umbilical cord blood as well as altered cell counts and transcriptional signatures of umbilical cord monocytes (16, 21). Taken together, these lines of evidence suggest that *in utero* exposure to maternal immune activation by SARS-CoV-2 infection may have potential downstream immune and inflammatory consequences for the fetus (1).

Studies reporting on differential offspring neurodevelopmental and cardiometabolic outcomes following *in utero* exposure to COVID-19 highlight the need to identify potentially protective factors (22–25). COVID-19 vaccines are safe and effective in preventing severe disease from COVID-19 in pregnant individuals and their neonates, who benefit from transplacental transfer of vaccine-derived antibodies for at least the first 6 months of life (26–28). Vaccination against COVID-19 with mRNA vaccines has been shown to alter the immune response to SARS-CoV-2 infection in non-pregnant populations (29–31). The objectives of this study were to: 1) evaluate the serum cytokine profile of the maternal immune response to SARS-CoV-2 infection, in pregnant individuals with and without prior COVID-19 vaccination, compared with uninfected pregnant controls and 2) to evaluate the cord serum cytokine signature in the setting of acute maternal SARS-CoV-2 infection at the time of delivery in the same groups. We hypothesized that the fetal immune response to maternal immune activation in SARS-CoV-2 would be blunted in vaccinated pregnancies.

## Materials and methods

### Participant selection and recruitment

Sixty-six pregnant individuals enrolled in the Massachusetts General Hospital COVID-19 biorepository between March 2020 and April 2022 were included in the study. Maternal sera were collected at the time of acute SARS-CoV-2 infection in 26 unvaccinated individuals and 21 previously vaccinated individuals. Cord sera were collected and analyzed from individuals with acute SARS-CoV-2 infection if delivery occurred during the infectious period, i.e. less than or equal to 14 days from positive test in asymptomatic individuals or from onset of symptoms in symptomatic individuals. Maternal and cord sera from 19 healthy mother-cord dyads at term without a history of SARS-CoV-2 infection during pregnancy and who tested negative for SARS-CoV-2 upon admission to the Labor and Delivery unit were analyzed as controls. Participant enrollment occurred during a period when universal SARS-CoV-2 testing was performed on admission to the Labor and Delivery unit, thus the SARS-CoV-2 status of all participants was known on admission for delivery. All participants provided informed consent. Identification of eligible individuals, participant recruitment strategies and enrollment procedures have been described in previous publications (17, 32). A flow chart describing study participants is presented in the Supplementary (**Supplementary Figure S1**).

Pregnant individuals were eligible for inclusion if they were diagnosed with SARS-CoV-2 infection or known to be negative for SARS-CoV-2 by nasopharyngeal swab RT-PCR. Maternal SARS-CoV-2 positivity was defined by a positive nasopharyngeal swab RT-PCR. Participants negative for SARS-CoV-2 on admission to Labor and Delivery were enrolled as a convenience sample, recruited on the same days as enrolled positive cases. Demographic and clinical outcomes data were abstracted from the electronic medical record using REDCap electronic data capture tools (33). COVID-19 disease severity was defined according to National Institutes of Health criteria (34). Additional information on inclusion and exclusion criteria are provided in the Supplementary (**Supplementary Figure S1**).

### Sample collection and determination of cytokine concentrations in maternal and umbilical cord blood

Maternal and umbilical cord blood samples were obtained by venipuncture into serum separator tubes (BD). Blood was centrifuged at 1000g for 10 minutes, aliquoted and stored at -80°C. The concentrations of 20 cytokines were measured using the ProcartaPlex Human Inflammation Panel 20-plex (ThermoFisher) (**Supplementary Table S1**). All standards and samples were run in duplicate. The standard curve for each analyte was determined using the ProcartaPlex Analysis App (ThermoFisher). Standards with less than 80% recovery or more than 120% recovery were omitted, consistent with recommended thresholds. Analytes that were undetectable in more than 30% of samples were excluded from further analysis. For the remaining analytes, the limit of detection (LOD) of the analyte divided by √2 was substituted for levels below the LOD. For values above the upper limit of quantitation, the upper limit of quantitation was directly substituted. Outliers (defined as Z-score greater than 2.5) were excluded. All concentrations are reported as pg/mL.

### Statistical analysis

Univariate differences in maternal analytes were assessed by Kruskal-Wallis test with Dunn’s posthoc testing of the three study groups: 1) participants with unvaccinated SARS-CoV-2 infection, 2) participants with vaccinated “breakthrough” SARS-CoV-2 infection, and 3) SARS-CoV-2 negative control groups. Analyte levels are reported as median [Interquartile Range (IQR)]. Spearman correlations between analytes within each group were determined and p-values adjusted for multiple comparisons using the Benjamini-Hochberg method. Spearman correlations of maternal to cord levels of each analyte were assessed in dyads who delivered during acute SARS-CoV-2 infection and in SARS-CoV-2 negative dyads. For multivariate analyses, maternal and cord analyte concentrations were log_2_-transformed to improve normality. The function *prcomp* in the R statistical language and environment (www.r-project.org, version 4.0.2) was used to calculate principal components (PC). The top three PC were tested for associations with sample type (maternal or cord) using linear regression modeling. This analysis also included vaccination status, infant sex, and exposure to labor as possible covariates, based on *a priori* assumption that these factors may impact cytokine/chemokine levels in the maternal-fetal dyad (35, 36). All statistical tests were two-tailed, and significance was inferred based on p<0.05. Statistical analyses were performed in R (version 4.0.2).

## Results

### Participant clinical characteristics

Sixty-six pregnant individuals were included, 26 with acute SARS-CoV-2 infection who were unvaccinated, 21 with acute SARS-CoV-2 who had previously received a primary COVID-19 vaccine series, and 19 uninfected pregnant controls (**Supplementary Figure S1**). No placentas examined had histopathologic or clinical evidence of SARS-CoV-2 placental infection and were thus not examined for SARS-CoV-2 viral RNA. Of the 26 unvaccinated individuals with SARS-CoV-2 infection, 4 (15%) had asymptomatic disease, 10 (38%) had mild disease, 2 (8%) had moderate disease, 9 (35%) had severe disease and 1 (4%) had critical disease. Of the 21 vaccinated individuals, 6 (28%) had asymptomatic disease, 12 (57%) had mild disease, 3 (14%) had moderate disease, and no individuals had severe or critical disease. Of the 21 vaccinated individuals, 14 received the BNT162b2 primary series, 6 received the mRNA-1273 primary series, and 1 individual received one dose of Ad26.COV2.S and one dose of mRNA-1273; 10 of the 21 vaccinated individuals had received a third mRNA vaccine dose prior to infection. There were no differences in maternal age, parity, race, ethnicity, body mass index (BMI), hypertensive disorders of pregnancy, neonatal sex, or labor between groups (**Table 1**). Placental pathological examinations were performed by clinical indication per institutional protocol and reported in the birthing individual’s medical record. No placentas examined had histopathologic evidence of SARS-CoV-2 infection, although SARS-CoV-2 RNA testing was not routinely performed during the study period due to prior observations that placental infection with SARS-CoV-2 is uncommon (17). Placental pathology findings were similar between groups (**Supplementary Table S3**). No newborns of the individuals included in this cohort tested positive for SARS-CoV-2.

**Table 1:** Clinical and demographic information of the cohort.

	Unvaccinated infection N=26	Vaccinated infection N=21	Uninfected controls N=19	P-value
COVID-19 severity (%)*				0.013
Asymptomatic	4 (15)	6 (29)	N/A	
Mild	10 (38)	12 (57)	N/A	
Moderate	2 (8)	3 (14)	N/A	
Severe	9 (35)	0 (0)	N/A	
Critical	1 (4)	0 (0)	N/A	
GA at positive SARS-CoV-2 test (weeks)	35 [30, 39]	34 [23, 38]	N/A	0.206
Maternal Age (median [IQR])	32 [28,34]	33 [31,35]	33 [30.36]	0.498
Parity (median [IQR])	1 [0,1]	1 [0,1]	1 [0,1]	0.932
Race (%)				0.282
Asian	0 (0)	2 (10)	0 (0)	
Black	2 (8)	2 (10)	2 (11)	
Other	8 (31)	3 (14)	2 (11)	
Unknown	3 (12)	0 (0)	1 (5)	
White	13 (50)	14 (67)	14 (74)	
Ethnicity (%)				0.078
Hispanic or Latino	12 (46)	4 (19)	4 (21)	
Not Hispanic or Latino	12 (46)	17 (81)	14 (74)	
Unknown/not reported	2 (8)	0 (0)	1 (5)	
BMI (median [IQR])	26 [25,30]	23 [21,29]	23 [21,26]	0.057
Preeclampsia/HDP (%)	3 (13)	2 (14)	2 (11)	1
Fetal sex (%)				
Female	9 (39)	8 (53)	10 (53)	0.663
Male	14 (61)	7 (47)	9 (47)	
Labor (%)	18 (78)	12 (80)	14 (74)	1
GA at delivery	39 [38, 40]	39 [39, 40]	39 [38, 41]	0.909
Days from SARS-CoV-2 diagnosis to sample collection (mean +/- SD)	3.9 (4.5)	10.5 (8.1)	N/A	0.002

GA, gestational age; BMI, body mass index; HDP, hypertensive disorder of pregnancy. *Severity defined by NIH criteria endorsed by the Society for Maternal Fetal Medicine.

### Differences in the maternal serum immune response to SARS-CoV-2 infection during pregnancy reflect engagement of cellular immunity in previously vaccinated individuals

**Figure 1:**
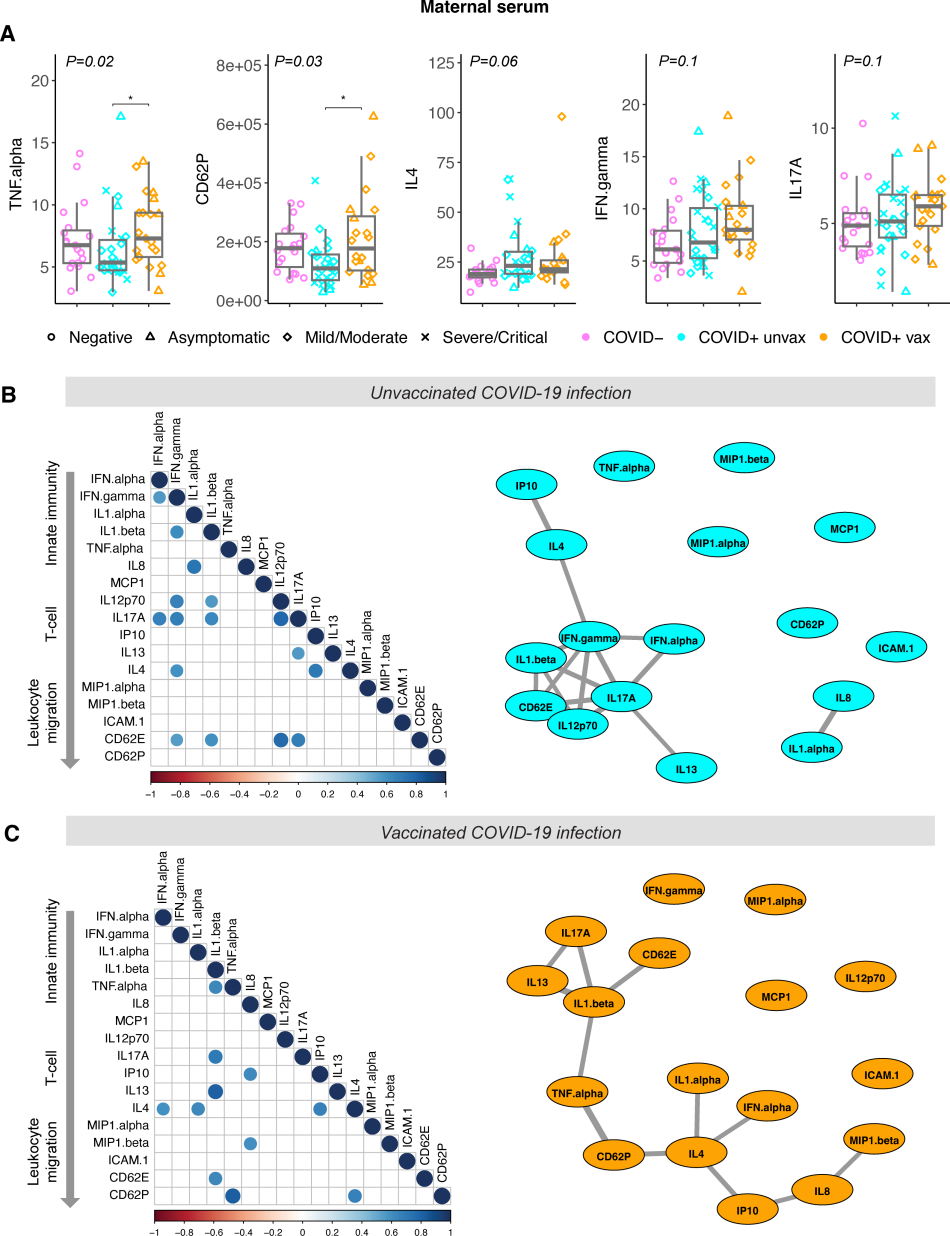
Cytokine levels in maternal sera from vaccinated and unvaccinated participants with maternal SARS-CoV-2 infection, and negative controls. COVID-19 severity as defined by NIH criteria indicated by shape. Unvax = unvaccinated; vax = received primary COVID-19 vaccine series prior to infection. **(A)** Analyte levels of maternal sera collected from unvaccinated (blue, N=26) and vaccinated (orange, N=21) participants during acute SARS-CoV-2 infection, and negative controls at delivery (purple, N=19). Concentrations reported as pg/mL. Boxplots indicate median and interquartile range. Group differences assessed by Kruskal-Wallis test (group P-value shown). Significant differences between groups on Dunn’s posthoc testing indicated by *P<0.05. **(B)** Correlation analysis of maternal serum analytes from unvaccinated participants (N=26) and **(C)** vaccinated participants (N=21). Left panel: dot plot of significant Spearman correlations (padj<0.05). Dot size and color indicate strength of correlation, with blue indicating positive and red indicating negative correlations. Right panel: network correlation plot with each analyte depicted as a node and positively-correlated analytes (padj<0.05) indicated by weighted gray lines.

Maternal sera were collected a median of 4 days [interquartile range (IQR): 1-11 days] from the onset of acute infection. Significantly higher maternal serum levels of TNFα (p=0.03) and soluble CD62P (p=0.03) were observed in vaccinated individuals with SARS-CoV-2 infection compared with SARS-CoV-2 infected but unvaccinated individuals (**Figure 1A**). Adjusting for sample collection timing in days using linear regression modeling had no effect on the significant findings of differences in CD62P and TNFα levels between vaccinated and unvaccinated groups [TNFα: effect of group, p=0.029, effect of collection time, p=0.46; CD62P: effect of group, p=0.023, effect of collection timing, p=0.81]). Maternal serum levels of IFNγ, IL4, and IL17A appeared higher in vaccinated individuals with SARS-CoV-2 infection compared to controls, however these differences did not reach statistical significance. No differences in other maternal serum cytokines/chemokines evaluated were observed between groups (**Supplementary Figure S2**).

To further assess the maternal inflammatory response to SARS-CoV-2 we next calculated Spearman correlations between serum analytes, in SARS-CoV-2-infected individuals with and without prior vaccination (**Figures 1B, C**). After adjustment for multiple comparisons, 16 significant positive correlations were observed between analytes in unvaccinated infection and 12 positive correlations in vaccinated infection, compared with only 1 positive correlation in negative controls (**Supplementary Figure S3**), as anticipated. Network plots depicting relationships between correlated analytes show tight correlations between interferons (IFNα and IFNγ) that are critical to the innate antiviral immune response and analytes associated with Th1 responses (IL12p70, IL1β, IL17A) in individuals without prior vaccination (**Figure 1B**). In contrast, the maternal cytokine response to vaccinated infection is not centered on interferons, but rather IL4, strongly associated with Th2 responses (**Figure 1C**). These findings suggest that SARS-CoV-2 infection occurring after vaccination may be more strongly associated with Th2-mediated responses in pregnant individuals.

### Analyses of cord cytokine levels does not indicate a coordinated fetal inflammatory response to maternal SARS-CoV-2 infection

Prior work suggests that a fetal immune response may be observed in the setting of maternal infection with SARS-CoV-2 that is linked to the degree of maternal immune activation and clinical disease severity (15, 21, 37) In contrast to what we observed in the maternal circulation in the setting of SARS-CoV-2 infection, univariate analyses of cord analytes demonstrated no significant differences in cord serum analyte levels between individuals with and without SARS-CoV-2 infection at the time of delivery, regardless of prior COVID-19 vaccination history, and negative controls, with the exception of MIP1α which was significantly lower in unvaccinated infection compared to negative controls (**Supplementary Figure S4**). Additionally, no significant correlations were noted between cord analytes within groups. These data support the lack of a specific, coordinated fetal immune response to maternal SARS-CoV-2 infection in either vaccinated or unvaccinated individuals.

**Figure 2:**
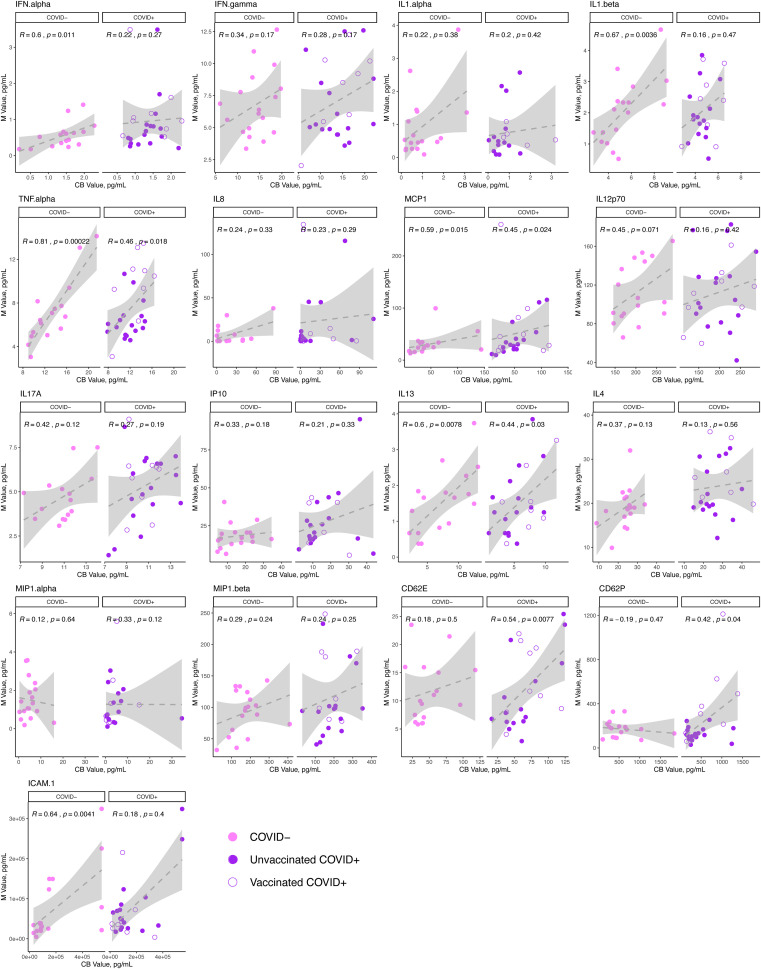
Correlation of cytokine analytes in the maternal and cord sera at delivery in acute SARS-CoV-2 infection and in SARS-CoV-2 negative controls. N=41 dyads: 23 maternal:cord pairs with acute maternal SARS-CoV-2 infection at delivery (purple, N=11 vaccinated, 12 unvaccinated),18 SARS-CoV-2 negative controls (pink). Maternal (M) analyte levels depicted on y-axis and cord blood (CB) levels on x-axis, with Spearman correlation and p-value indicated on plots. Open circles = vaccinated “breakthrough” infection.

To further assess the potential connection between maternal and cord cytokine responses to maternal SARS-CoV-2 infection, we next assessed Spearman correlations of analytes between paired maternal and cord samples obtained at delivery in individuals with acute SARS-CoV-2 infection (delivery occurring within 14 days of SARS-CoV-2 diagnosis), and uninfected controls. The results of these analyses are depicted in **Figure 2**. We identified that while maternal and cord levels of IL13, TNFα, and MCP1 were significantly positively correlated to each other in acute SARS-CoV-2 cases at delivery, these analytes were also correlated in healthy, SARS-CoV-2 negative maternal: cord dyads. Maternal and cord levels of CD62E and CD62P – leukocyte adhesion molecules that are expressed by activated endothelium and known to be elevated in some inflammatory pathologies – were positively correlated in SARS-CoV-2 cases but not controls, indicating that maternal endothelial activation may be related to fetal endothelial activation in SARS-CoV-2 cases, an effect not observed in healthy controls (38–40).

### Maternal cytokine profiles in the setting of acute SARS-CoV-2 infection at delivery are distinct from cord profiles and characterized by COVID-19-associated biomarkers

**Figure 3:**
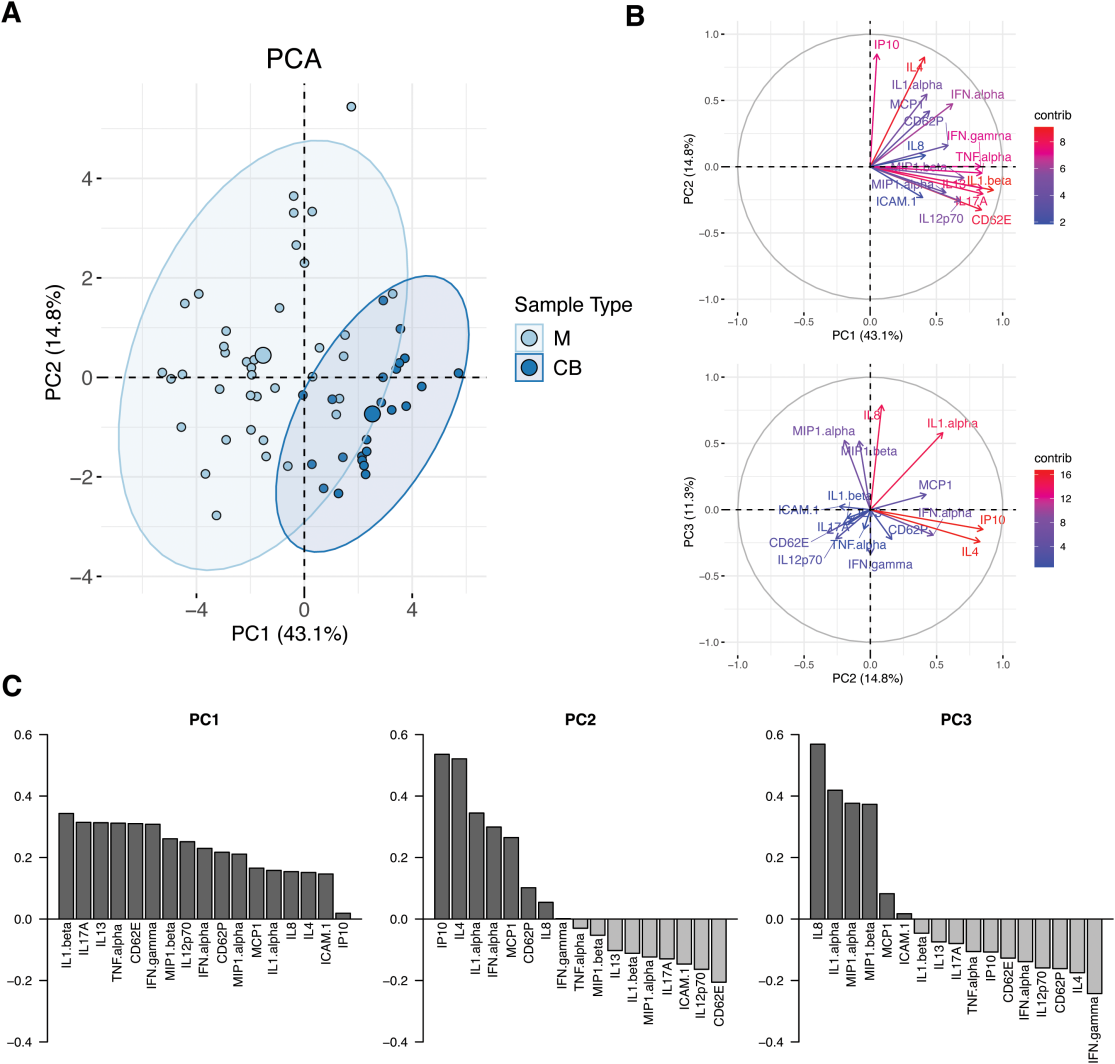
Principal component analysis (PCA) of maternal and cord analyte levels during acute maternal SARS-CoV-2 infection. **(A)** PCA plot of samples obtained during acute maternal SARS-CoV-2 infection. Cord samples were obtained following delivery occurring during acute maternal SARS-CoV-2 infection; no newborns tested positive for SARS-CoV-2 after birth. M, maternal blood (N=47, light blue). CB, cord blood (N=23, dark blue). Ellipses show 95% CI of sample distribution. **(B)** Contribution of analytes to PC1 *vs* PC2 (top) and PC2 *vs* PC3 (bottom). **(C)** Analytes with highest contributions to PC1, PC2 and PC3.

To build an integrated view of the serum inflammatory profile of the maternal-fetal dyad in the setting of acute maternal SARS-CoV-2 infection and to simultaneously handle the multiple correlated analytes, we used Principal Component Analysis (PCA) to resolve covariation between analytes into independent components (**Figure 3**). Separation of maternal and cord sera was observed along the first two PCs, which accounted for 43.1% and 14.8% of sample variation, respectively, indicative of overall distinct immune profiles between maternal and cord samples at the time of acute maternal infection (**Figure 3A**). The contribution of individual cytokines to the first 3 principal components (PC1-3) of the model are shown in **Figure 3B**. The top 5 cytokines contributing to the construction of PC1 are IL1β, IL17A, IL13, TNFα, and CD62E, whereas IP10 (CXCL10), IL4, IL1α, IFNα and MCP1 define PC2 (**Figure 3C**). Compared to maternal samples, cord samples clustered positively in PC1 – defined by increases in markers of both classic pro-inflammatory (IL1β, IL17A, TNFα) and anti-inflammatory (IL13) responses. Maternal samples were positive in PC2, defined by significant contribution from IP10 (CXCL10) and IL4, factors that have been previously identified as serum biomarkers of COVID-19 severity (41, 42).

Linear regression models of the first 3 PCs, which describe 69% of the variation in the dataset, were built to assess the contribution of clinical covariates to sample variation: sample source (maternal or cord), history of vaccination prior to infection, infant sex, and presence of labor. PC1 and PC2 were positively associated with cord sample (p<0.001 and p<0.01, respectively); presence of labor was associated with PC3 (p<0.05), driven by chemokines IL8, MIP1α and MIP1β. Prior vaccination and infant sex were not correlated with the first 3 PCs of the model. Timing of infection to sample collection had no impact on PC1 or 2 but it did have a small but significant impact on PC3 (p<0.05). Taken together these findings suggest that maternal and cord cytokine/chemokine profiles in the setting of SARS-CoV-2 are distinct and not significantly impacted by maternal vaccination status or infant sex; exposure to labor had a minor impact on chemokines represented by PC3, which itself represented only 11% of variation in the dataset. Details of models are available in **Supplementary Table S2**.

## Discussion

In this cohort study of 66 pregnant individuals, we identified that SARS-CoV-2 “breakthrough” infection (BTI), i.e. infection with SARS-CoV-2 in a previously vaccinated individual, was associated with a more robust maternal cytokine signature compared with unvaccinated infection. These findings resonate with recent observations of a similar pattern of elevated proinflammatory cytokines despite milder clinical disease in BTI compared with infection in unvaccinated pregnant individuals (43). In this work by Borsetti and co-authors, maternal BTI was also associated with greater complement of memory T-cells, higher anti-SARS-CoV-2 IgG, and enhanced transplacental antibody transfer compared with unvaccinated infection, implying a robust immune response. Likewise, vaccine-primed immune memory in nonpregnant cohorts results in concerted T- and B-cell responses (44, 45) In our study of the maternal peripheral cytokine signature of BTI in pregnancy, network correlation analyses suggest differences in the immune signature that may reflect bias of Th17 and Th2 responses in BTI, compared with interferon-driven Th1 responses in unvaccinated infection.

We found that cord cytokine levels did not differ significantly in dyads with SARS-CoV-2 infection at delivery and negative controls, regardless of maternal vaccination status. These data are concordant with a recent systematic review and meta-analysis of studies assessing cytokines in maternal-infant dyads following SARS-CoV-2 infection during pregnancy, which identified no significant differences in cord cytokine levels between cases and controls (46, 47). Although differences in clinical characteristics, samples studied, and analytic techniques complicate direct comparisons, in general, studies that have identified differences in cord inflammatory profiles in the setting of maternal SARS-CoV-2 infection were primarily assessing infections in unvaccinated individuals, which may have a different inflammatory impact (15, 21, 37).

Despite observing no clear fetal inflammatory response to maternal infection, many cord cytokine levels were found to be positively correlated with maternal levels in all groups at delivery. Upon analysis of clinical covariates, we found that exposure to labor had a small but significant impact on variation in dyad cytokine levels. These data are consistent with prior reports that labor and delivery itself may be reflected in transient inflammatory responses in the maternal and fetal sera (48). Such transient cytokine elevations may be distinct from the more prolonged elevations in maternal pro-inflammatory cytokines noted in acute and even convalescent SARS-CoV-2 infection (21, 49). When assessing cytokine profiles in the setting of acute maternal SARS-CoV-2 infection, however, we found that maternal and fetal cytokine and chemokine profiles were distinct, supporting the concept that the fetus may be relatively shielded from the maternal immune activation observed in response to SARS-CoV-2.

One notable exception we identified was in levels of CD62E (E-selectin) and CD62P (P-selectin), both of which were positively correlated between mother and fetus in the setting of SARS-CoV-2 infection and were not correlated in SARS-CoV-2 negative control dyads. Future studies investigating the impact of maternal SARS-CoV-2 infection on the fetal endothelium may be relevant, particularly in light of evidence from our group that *in utero* exposure to maternal SARS-CoV-2 may impact offspring cardiovascular and metabolic programming (22, 25, 50).

A key strength of our study is the inclusion of an uninfected pregnant comparator group balanced for labor and fetal sex, which allows for interpretation of the maternal cytokine response to SARS-CoV-2 against the known dynamic changes in the inflammatory proteome that occur in the late third trimester and at delivery (51). We observed minimal differences in peripheral cytokine levels between the uninfected, predominantly laboring uninfected controls and individuals with unvaccinated infection, which resonates with the observation that peripheral inflammatory responses to SARS-CoV-2 in vaccine-naïve pregnant individuals is relatively subdued when compared with those of non-pregnant individuals (15). Prior work has also demonstrated the importance of balancing for fetal sex in the assessment of maternal peripheral cytokines, as individuals pregnant with a male fetus have greater proinflammatory/proangiogenic profiles at baseline compared to individuals pregnant with a female (52). An important limitation to acknowledge is the lack of serial samples in infected individuals, which precluded evaluation of whether an initial robust pro-inflammatory response associated with BTI extinguished more rapidly than the potentially chronic pro-inflammatory response associated with unvaccinated infection (21). The reliance on samples collected from participants hospitalized for clinical disease or during the delivery hospitalization also precluded the ability to standardize sample collection across uniform time points.

In conclusion, we identified a more robust pro-inflammatory profile in the maternal cytokine response to SARS-CoV-2 infection in pregnancy in individuals with prior vaccination compared to unvaccinated pregnant hosts. Differences in maternal serum cytokine profiles may reflect vaccine-mediated priming of the immune system that protects against severe disease and may enhance dyad immunity. A fetal inflammatory response specific to maternal SARS-CoV-2 infection was not identified in the umbilical cord sera of either vaccinated or unvaccinated groups. Taken together, these findings can reassure pregnant individuals that while vaccination is associated with a robust initial maternal inflammatory response upon reinfection, the fetus appears to be relatively shielded from the pro-inflammatory impact of maternal infection.

## Data Availability

The raw data supporting the conclusions of this article will be made available by the authors, without undue reservation.
