# Supplementary material for: Maternal-fetal cytokine profiles in acute SARS-CoV-2 “breakthrough” infection after COVID-19 vaccination
Source: Front Immunol. 2025 Jan 8;15:1506203. doi: 10.3389/fimmu.2024.1506203 (PMC11750656; doi:10.3389/fimmu.2024.1506203)
Supplement: Supplementary file 1 [file DataSheet1.docx]

FIGURE S1.


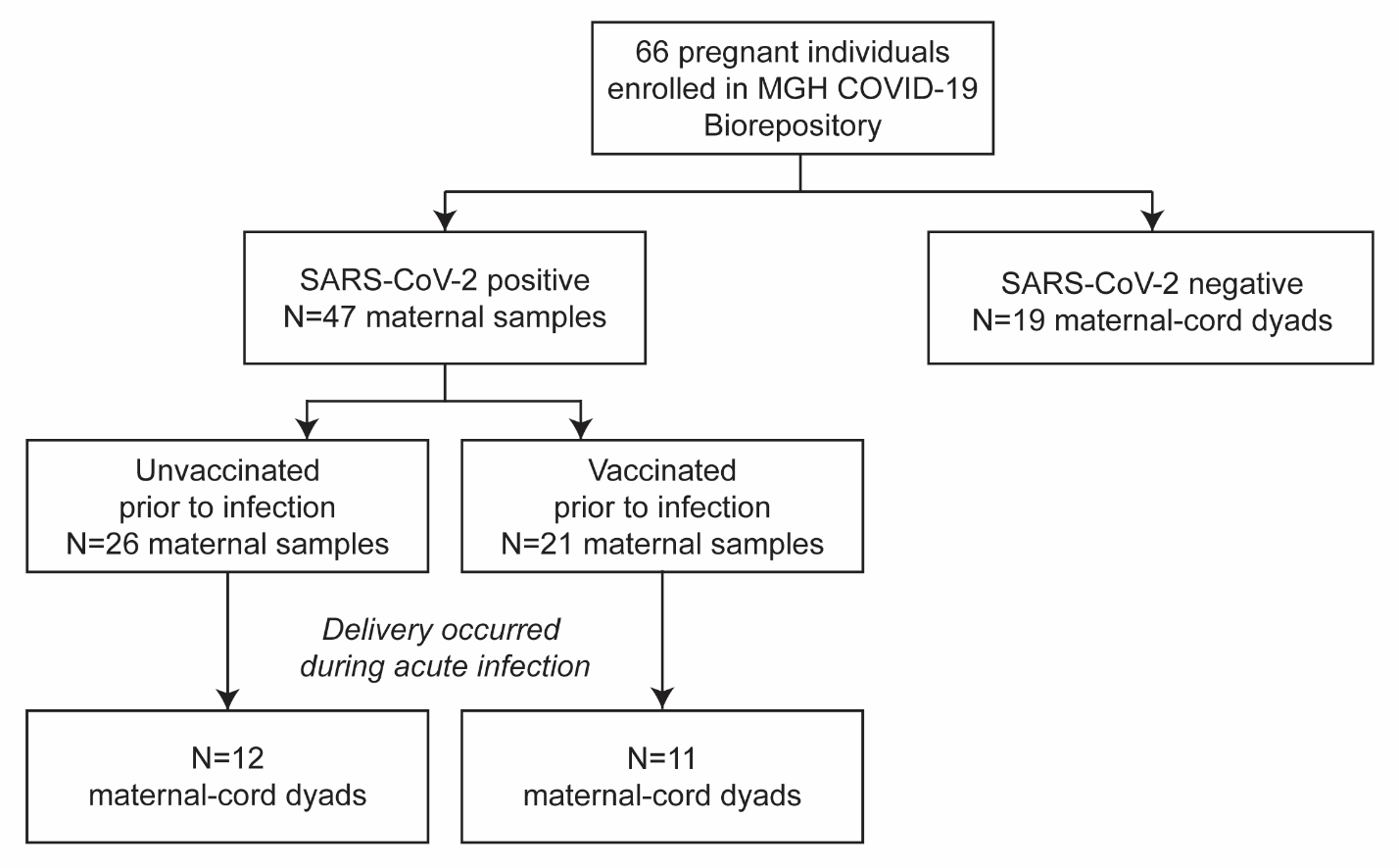


**Figure S1. Diagram of study cohort and design.** Pregnant participants were enrolled in the Massachusetts General Hospital (MGH) COVID-19 biorepository at the time of hospitalization for a COVID-19 related illness or at the delivery hospitalization (N=66). Inclusion criteria: (1) 18 years of age or older, (2) able to provide informed consent or with a healthcare proxy able to do so, (3) diagnosed with, or at risk for, SARS-CoV-2 virus infection. Exclusion criteria: known fetal anomaly, multiple gestation, history of acute chorioamnionitis at delivery. Participants were not excluded on the basis of placental pathology results. All participants were tested for the presence of SARS-CoV-2 by RT-PCR of nasopharyngeal swab upon hospitalization. Given the study’s primary focus on COVID-19 in pregnancy, contemporaneous controls were enrolled as a convenience sample, from individuals presenting to Labor and Delivery for care on the same days as enrolled cases. Maternal and cord sera were collected from SARS-CoV-2 negative controls at the delivery hospitalization if they had no history of prior SARS-CoV-2 infection during pregnancy. Maternal sera were collected from 47 pregnant individuals with SARS-CoV-2 during the acute infectious period (<14 days from onset of symptoms or positive SARS-CoV-2 test in asymptomatic individuals). If delivery occurred during acute infection, cord sera were collected and analyzed. Individuals were considered unvaccinated if they had no history of receiving a COVID-19 vaccine prior to SARS-CoV-2 infection. Individuals were considered vaccinated if they had completed a primary COVID-19 vaccine series prior to the SARS-CoV-2 positive test.

FIGURE S2.


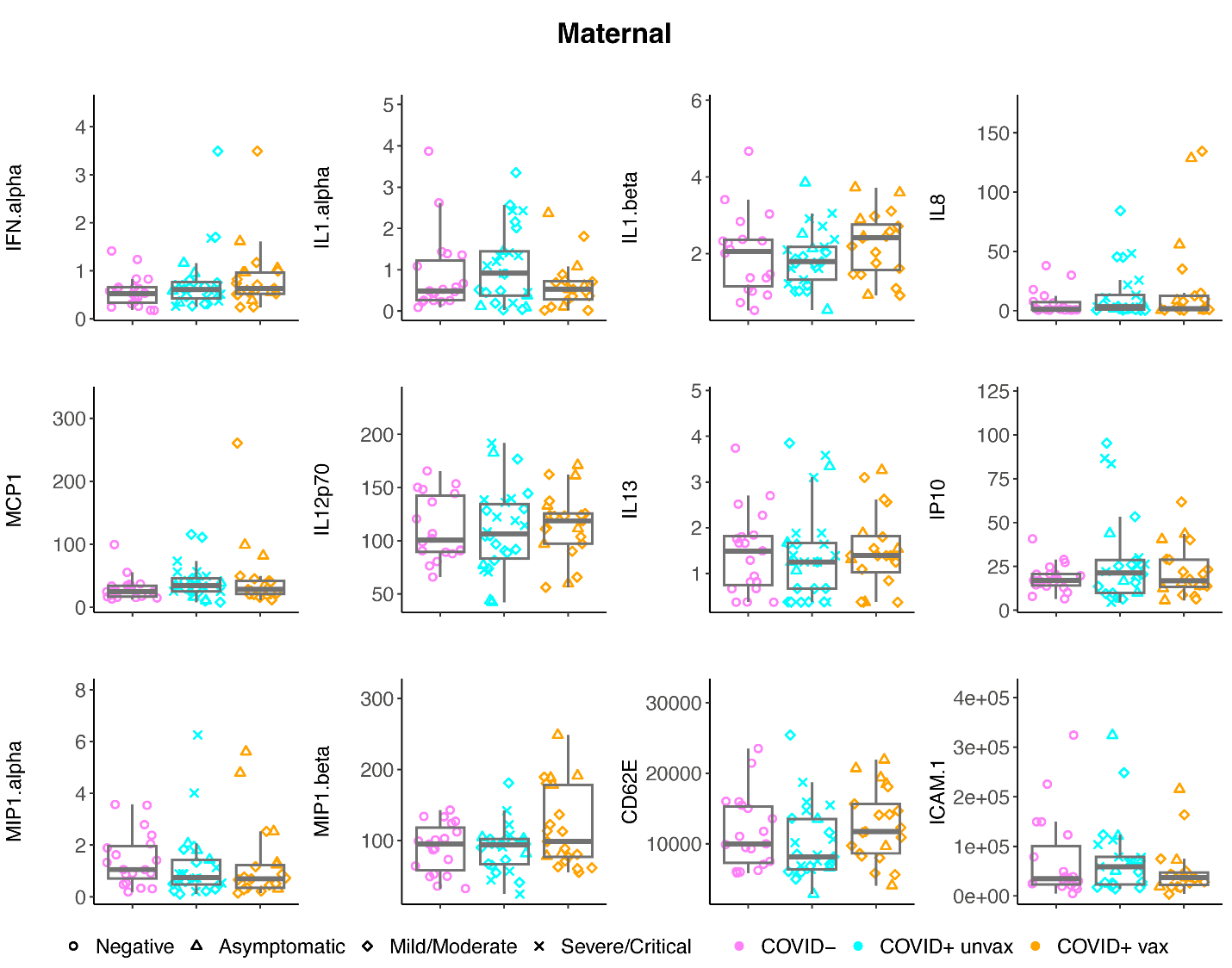


**Figure S2. Maternal analyte levels from vaccinated and unvaccinated participants with SARS-CoV-2 infection, and SARS-CoV-2 negative controls.** COVID-19 severity as defined by NIH criteria indicated by shape. Maternal sera collected from participants in unvaccinated (blue, N=26) and vaccinated (orange, N=21) participants during acute SARS-CoV-2 infection, and negative controls at delivery (purple, N=19). Concentrations reported as pg/mL. Boxplots indicate median and interquartile range. Group differences assessed by Kruskal-Wallis test.

FIGURE S3.


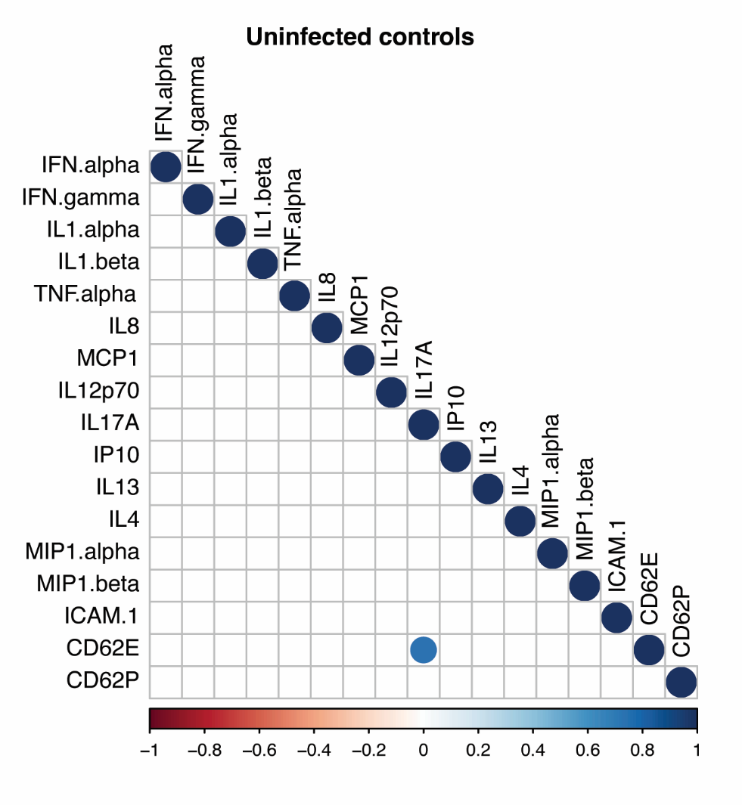


**Figure S3. Correlation of maternal analyte levels from negative controls at delivery.** Dot plot of significant Spearman correlations (Padj<0.05), N=19.

FIGURE S4.


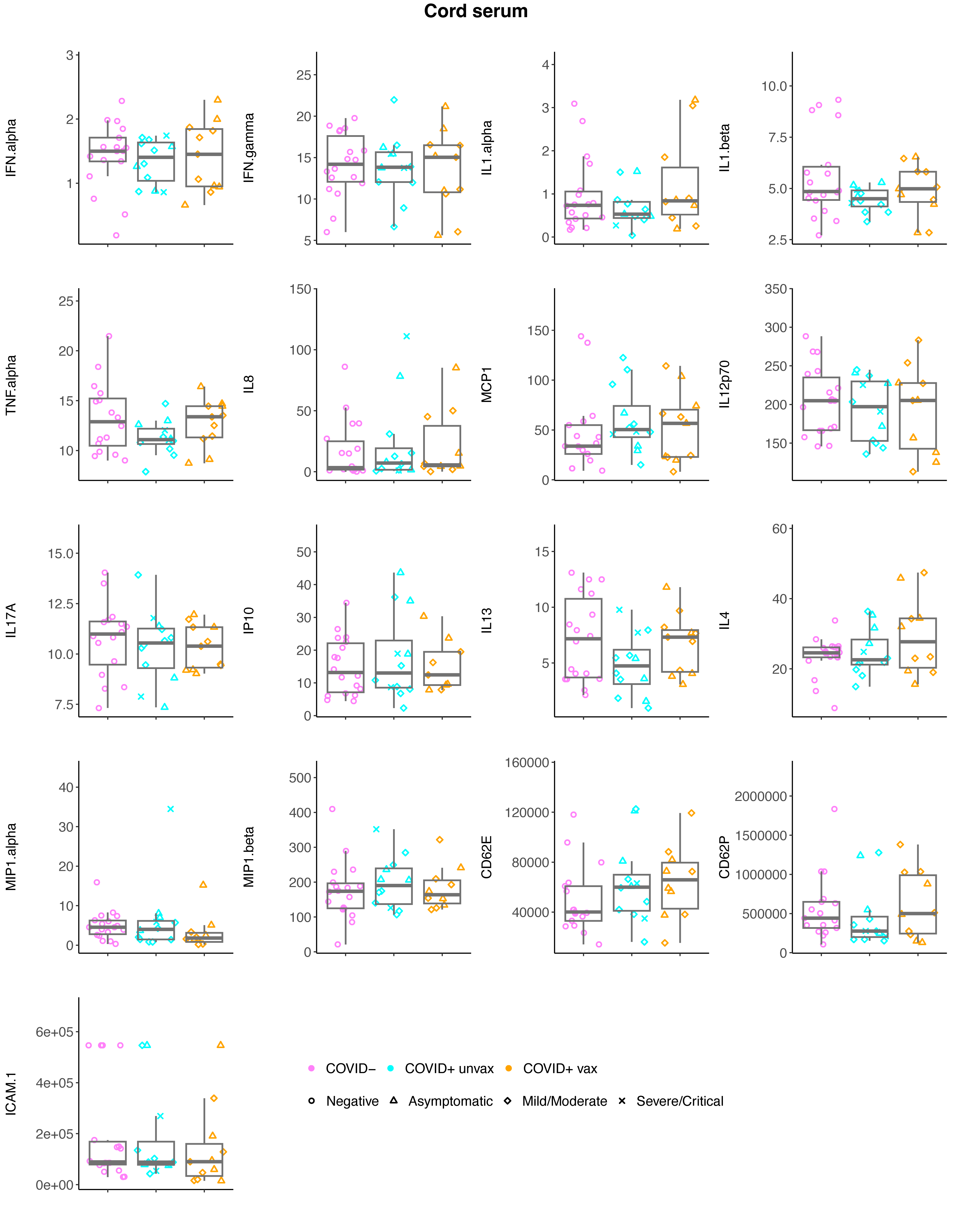


**Figure S4.** **Analyte levels in cord sera following delivery in the setting of acute maternal SARS-CoV-2 infection, and negative controls.** Analyte levels in cord sera collected from unvaccinated (blue, N=12) and vaccinated (orange, N=11) participants with acute maternal SARS-CoV-2 infection at delivery (less than 14 days from positive test). Cord samples were obtained following delivery occurring during acute maternal SARS-CoV-2 infection; no newborns tested positive for SARS-CoV-2 after birth. Negative controls from healthy term deliveries (purple, N=18). Concentrations reported as pg/mL. Boxplots indicate median and interquartile range. Group differences assessed by Kruskal-Wallis test.

**Table S1. List of 20 inflammatory cytokines, chemokines, and cell adhesion molecules profiled.**

| **Cytokines** | **Chemokines** | **Cell adhesion/ response to inflammation** |
| --- | --- | --- |
| GM-CSF  IFN-α  IFN-γ  IL-1β  IL-1α  IL-4  IL-6  IL-8  IL-10  IL-12p70  IL-13  IL-17A  TNF-α | IP-10 (CXCL10)  MCP-1 (CCL2)  MIP-1α (CCL3)  MIP-1β (CCL4) | ICAM-1  CD62E (E-selection)  CD62P (P-selectin) |

**Table S2. Linear regression modeling of top 3 PCs by clinical covariates.**

| **Model** | **Term** | **Estimate** | **Std. Error** | **Statistic** | **P-value** |
| --- | --- | --- | --- | --- | --- |
| **PC1** | **Cord sample** | **3.86651** | **0.49484** | **7.814** | **<0.001** |
| PC1 | Prior vaccination | 0.83282 | 0.52179 | 1.596 | 0.11620 |
| PC1 | Labored | 0.69508 | 0.61815 | 1.124 | 0.26571 |
| PC1 | Male infant sex | -0.39032 | 0.48859 | -0.799 | 0.42780 |
| PC1 | Days from infection to collection | -0.05111 | 0.03530 | -1.448 | 0.15334 |
| **PC2** | **Cord sample** | **-1.23405** | **0.40513** | **-3.046** | **0.00356** |
| PC2 | Prior vaccination | 0.40152 | 0.42719 | 0.940 | 0.35137 |
| PC2 | Labored | -0.04945 | 0.50609 | -0.098 | 0.92252 |
| PC2 | Male infant sex | -0.17712 | 0.40002 | -0.443 | 0.65966 |
| PC2 | Days from infection to sample collection | -0.04198 | 0.02890 | -1.452 | 0.15211 |
| PC3 | Cord sample | -0.42963 | 0.35635 | -1.206 | 0.2331 |
| PC3 | Prior vaccination | 0.16519 | 0.37576 | 0.440 | 0.6619 |
| **PC3** | **Labored** | **1.09260** | **0.44516** | **2.454** | **0.0173** |
| PC3 | Male infant sex | -0.05674 | 0.35186 | -0.161 | 0.8725 |
| **PC3** | **Days from infection to sample collection** | **-0.05446** | **0.02542** | **-2.142** | **0.0366** |

**Table S3. Results of pathological examinations of placentas in the study cohort**

|  | Unvaccinated  SARS-CoV-2 infection  N=22 | Vaccinated  SARS-CoV-2 infection  N=19 | Uninfected controls N=10 |
| --- | --- | --- | --- |
| Acute chorioamnionitis (%) | 5 (22.7%) | 2 (10.5%) | 2 (20%) |
| Villitis (%) | 4 (18.2%) | 5 (26.3%) | 3 (30%) |
| Thrombus (%) | 5 (22.7%) | 5 (26.3%) | 4 (40%) |
| Infarct (%) | 2 (9.1%) | 2 (10.5%) | 2 (20%) |
| Maternal vascular malperfusion (%) | 2 (9.1%) | 2 (10.5%) | 2 (20%) |
| Fetal vascular malperfusion (%) | 6 (27.3%) | 2 (10.5%) | 2 (20%) |
| Placental Weight (median [IQR]) | 400.0 [340, 465] | 452.0 [394, 494] | 448.0 [378, 525] |
| Placental weight  <10^th^ percentile | 8 (36.4%) | 11 (57.9%) | 6 (60%) |
